# Supplementary material for: Splice-Junction-Based Mapping of Alternative Isoforms in the Human Proteome
Source: Cell Rep. Author manuscript; Available in PMC 2020 Jan 15. (PMC6961840; doi:10.1016/j.celrep.2019.11.026)
Supplement: 2 [file NIHMS1546469-supplement-2.zip › DF1/PXD010154/Testis/Testis_16_NCOR1_YSVPPVLQPAPHQVITNLPEGVR.pdf]

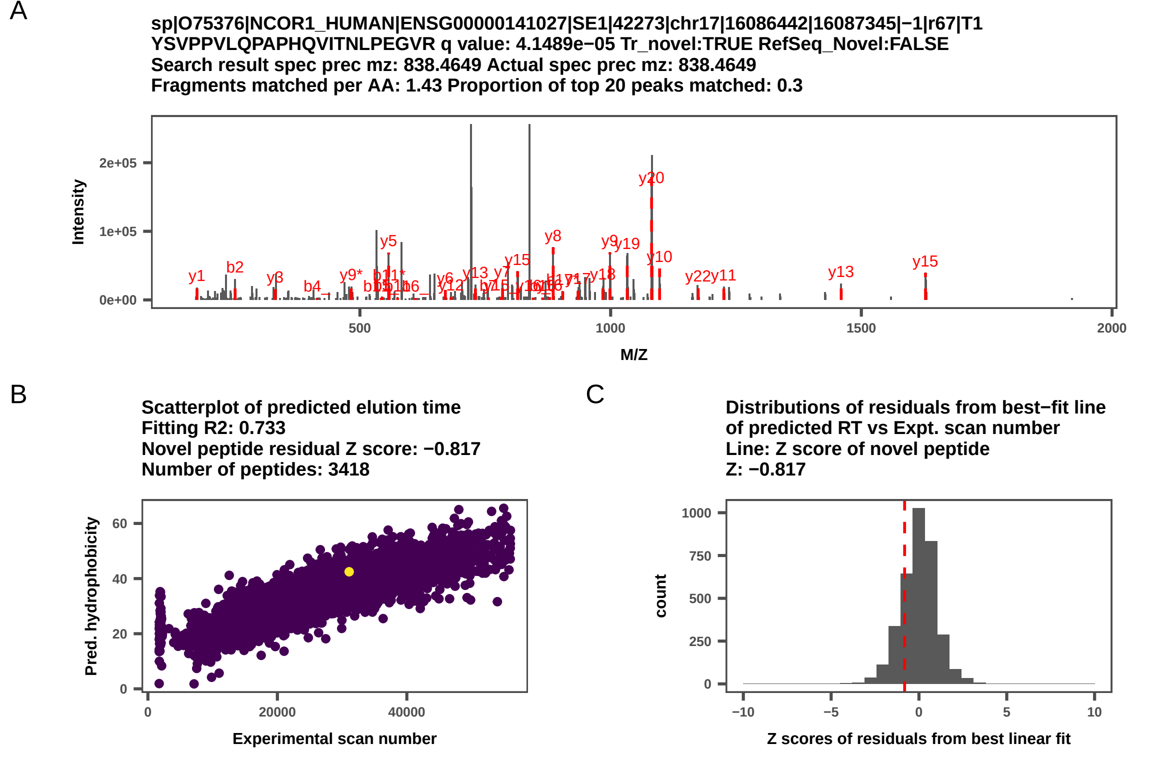
A
sp|O75376|NCOR1_HUMAN|ENSG00000141027|SE1|42273|chr17|16086442|16087345|−1|r67|T1
YSVPPVLQPAPHQVITNLPEGVR q value: 4.1489e−05 Tr_novel:TRUE RefSeq_Novel:FALSE
Search result spec prec mz: 838.4649 Actual spec prec mz: 838.4649
Fragments matched per AA: 1.43 Proportion of top 20 peaks matched: 0.3
Intensity
2e+05
1e+05
0e+00
y1
b2
y3
b4
y5
y9*
b11*
y6
y12
y13
y7
y15
y8
y9
y19
y20
y10
y18
y22
y11
y13
y15
500
1000
1500
2000
M/Z
B
Scatterplot of predicted elution time
Fitting R2: 0.733
Novel peptide residual Z score: −0.817
Number of peptides: 3418
Pred. hydrophobicity
60
40
20
0
0
20000
40000
Experimental scan number
C
Distributions of residuals from best−fit line
of predicted RT vs Expt. scan number
Line: Z score of novel peptide
Z: −0.817
count
1000
750
500
250
0
−10
−5
0
5
10
Z scores of residuals from best linear fit
